# Evaluation of the Effects of Charged Amino Acids on Uncontrolled Seizures

**DOI:** 10.1155/2015/124507

**Published:** 2015-07-09

**Authors:** Hossein Ali Ebrahimi, Saeed Ebrahimi

**Affiliations:** Neurology Research Center, Kerman University of Medical Sciences, Kerman, Iran

## Abstract

*Introduction*. Epilepsy is one of the most common diseases of the central nervous system. The prevalence of epilepsy throughout the world is 0.5 to 1%, and the same rate is 7.8 per 1000 in Kerman. Almost 20 to 30% of epileptic patients do not respond properly to common medications. The present study investigated patients who did not respond to common and, even in some cases, adjuvant therapies, with two seizures or more per week, regardless of the type of the inflicted epilepsy. *Methodology*. The participants of the present double-blind study were randomly selected into three 10-member groups of uncontrolled epileptic patients (arginine, glutamic acid, and lysine). The patients used amino acid powder dissolved in water (three times the daily need) every day for two weeks before breakfast. The number of seizures was recorded one week prior to commencing amino acid use, as well as the first and the second weeks subsequent to use. *Results*. A total of 32 patients were studied in three groups. The decline rates of seizures were 53%, 41%, and 13%, and the *P* value was 0.013, 0.027, and 0.720, respectively. *Conclusion*. Administration of the charged amino acids, arginine, and glutamic acid can decrease the seizures of patients suffering from uncontrolled epilepsy.

## 1. Introduction

Epilepsy is one of the most prevalent diseases of the central nervous system with a prevalence of 0.5 to 1 percent of the world population [1]. An estimated 50 million individuals suffer from epilepsy worldwide [2, 3]. According to the conducted studies, 7.8 per thousand individuals suffer from epilepsy in Kerman, Iran, and are in need of anticonvulsant medication [4]. Despite the considerable advances in the treatment of epilepsy, especially production of new, more tolerable anticonvulsants, 20 to 30% of the patients suffer from uncontrolled seizures, referred to as medically resistant [5–8].

Besides uncontrolled seizures, side effects of the administered anticonvulsants are important in treating the mentioned patients, which may hinder the administration of certain anticonvulsants. Unavailability as well as unreasonable prices of some anticonvulsant drugs may also add to the problems. Another impediment is the psychological complications caused by the long-term use of anticonvulsants (besides their side effects) causing untimely discontinuation of the drug and failed treatment [9, 10].

In addition to medical therapies, adjuvant therapies, like avoiding facilitating factors (light, stress, and sleep withdrawal), as well as psychiatric therapies, have received attention by scholars [11–14].

Among the challenging issues is the diet, concerning which different views have been reported in terms of the efficacy and positive or negative influence of certain foods. Ketogenic diet has long been proposed and used by the medically resistant patients for many years. Atkins diet, which leads to ketosis, has also received attention [15–17]. Consumption of foods containing protein chains has been proposed as well [18].

A study on rat showed that charged amino acids are capable of elongating the seizure latency induced by intraperitoneal injection of pentylenetetrazole (PTZ), as well as shortening seizure duration [19].

Charged amino acids are of two types, acidic or basic, depending on their Ph in the physiological environment. Glutamic and aspartic amino acids are acidic, while arginine and lysine are basic. Besides being a stimulator neurotransmitter, glutamic acid, as an acidic charged amino acid, plays a great role in the brain metabolism [20, 21], present in different brain parts and synapses, which functions via ionotropic or metabotropic receptors [22–25]. Another property of the glutamate brain metabolism is its close association with the citric acid cycle and energy metabolism [24, 26].

Arginine is a basic amino acid, and according to extensive studies of 1950 through to 1970, it is considered as a nonessential amino acid for the health of adults [27] and an essential amino acid for the growth of human beings and animals [28, 29]. However, upon the inflection of diseases, as well as physical traumas, it turns into an essential amino acid [30]. The sources of free arginine within the body are dietary protein, endogenous synthesis, and turnover of body proteins [31–34].

The importance of lysine is due to its involvement in the collagen construction. It is also among the most important components of the conjunctival tissue, essential for growth. Lysine is important for carnitine synthesis. The daily amount of lysine intake is 40–180 mg per kilogram of body weight; however, there have been reports of up to 300–400 mg intake [35].

The present study investigated the effects of charged amino acids on the seizures of uncontrolled epilepsy patients.

## 2. Methodology

As an interventional study, the present research investigated epilepsy patients who referred to the Neurology Clinic of Shafa Medical Center a subsidiary of Kerman University of Medical Sciences, Kerman, Iran, as well as private clinics, regardless of the type of the inflicted epilepsy, and who, despite using multiple anticonvulsant regimens, still experienced seizures, though drug side effects were observable. To allow for a better assessment, patients with at least two seizures in a week entered the study.

Prior to inclusion, the entire necessary examinations including self-report, follow-ups, EEG, and imaging were conducted by a neurologist to ensure the presence of epilepsy. It was ensured that the patients who entered the study, or their companions, were made aware of the fact that the mentioned substances have never been administered for such cases and also the possibility that it was probably the first time they, or their patients, were going to have an examination. Subsequently the participants declared their informed consent and were included. The patients were allowed to leave the program whenever they demanded.

It should be mentioned that use of these substances is embedded in humans daily diet, and furthermore there have been no reports concerning associated complications, and they do not seem to induce certain disorders, as the toxic dosage of the utilized substances is 50–500 times the daily required amount [36].

Patients who suffered from diseases other than epilepsy (a primary brain disease) were refused to enter the study. Pregnant women and breastfeeding mothers [37–39], psychotic patients [38, 39], and patients suffering from infections, particularly viral infections, were excluded.

Duration of the treatment was two weeks. The patients or their companions were asked to record the number of the seizures. The number of the seizures was recorded one week prior to amino acid use. The patients used amino acid powder dissolved in water, three times the daily need, and every day before breakfast. The number of seizures was once again recorded one week later. In case any complications (nausea, vomit) were reported, the patient was excluded from the study.

Charged amino acids are of 4 types, three of which were investigated in the present study. Aspartic acid was left out as it could not be procured and since it can replace glutamic acid in the body.

The amount of administered glutamic acid was a daily dosage of 10 grams for adults and 2 grams for children under 2 years of age. The administered lysine was a daily dosage of 6 grams for adults and 90 mg for children per kilogram of body weight. And arginine was administered at a daily dosage of 15 grams for adults and 240 mg for children per kilogram of body weight [40].

The patients were divided into three groups of 10 (with regard to the available patients) and each group was put on one of the foregone amino acids. The participants were randomly selected and appointed to a group. Allocating an amino acid to one of the three groups was double blinded; that is, neither the patient nor the attending physicians were aware of the type of the amino acid administered. The pertinent amino acids were already placed in packets of 14, handed to the patient bearing only a number. The packaging was carried out prior to prescription; as a result, the treating physicians were unaware of the contents.

The collected data went through statistical analysis. The results were compared to the recorded data prior to commencement of the study and the results from the three groups were compared. The recorded findings from the two previous stages as well as one and two weeks subsequent to administration as well as the number of seizures in the three groups were compared.

## 3. Results

A number of 45 patients were studied, of whom 13 individuals were excluded due to experiencing nausea and vomit and refusing cooperation resulting from tastelessness or undesirable taste of arginine, which left us with the remaining cooperative 32 patients.

Table 1 shows the number of patients in each group along with the average weekly seizures, one week prior to amino acid administration, and the first and second weeks after treatment commencement, respectively, as well as the proportion of the seizures of the second week compared to those of the week leading to treatment.

Sex distribution of the patients, according to the used amino acid, is presented in Table 2. There were no significant differences between the three study groups (*P* = 0.28).

Age distribution of the patients, according to the used amino acid, is presented in Table 3. There were no significant differences between the three study groups (*P* = 0.7).

Table 4 portrays the frequency of the different types of uncontrolled epilepsies in terms of the administered amino acid.

## 4. Discussion

Amino acids are vital for brain activity, the general deficiency of which may cause apathy, impaired concentration, loss of motivation, insomnia, mood disorders, anxiety, depression, self-harm, and aggression. They are essential in the construction of necessary and important proteins for the production of neurotransmitters required for the nervous system. Poor diets may lead to amino acid shortage and give rise to a genetic potentiality for amino acid deficiency [41].

Use of amino acids is greatly influential on paroxysmal disorders and the psychopathologic components of epilepsy. As for infants, administration of amino acids contributes to the mental and verbal development. The positive effects of amino acids in children will take 7–10 days to function, which may remain for a long time despite discontinuation. Amino acids may, furthermore, be effective in changing generalized epilepsy into focal epilepsy, as well as decreasing the frequency of epileptic seizures [42].

Acid glutamic managed to reduce the seizure frequency (*P* = 0.027) in uncontrolled epilepsy patients by more than 40% (Table 1). Studies conducted in the past few decades are evidence for the role of glutamic acid in epilepsy [43], which depends on the glutamate receptors, some of which have intensifying effects (type 1) and some other anticonvulsant effects (types 2 and 3) [44]. Effects of these receptors are mainly corrective, rather than directly stimulant or prohibitory [45]. It is assumed that oral amino acids may reinforce the roles of receptors in the natural course and, accordingly, improve controlling epileptic seizures.

Arginine could also reduce the seizure frequency (*P* = 0.013) in uncontrolled epilepsy patients by 53% (Table 1). L-arginine is an amino acid commonly sold in supplement form and obtained naturally in the diet. L-arginine-rich foods include plant and animal proteins, such as dairy. Arginine is a nonessential amino acid, the internal production of which is, however, insufficient and needs to be compensated for orally [30, 46, 47]. Arginine regulation is a subject of the extent of oral use and its internal production; hence, arginine needs to be viewed as a semiessential amino acid [31]. The ultimate metabolism of arginine leads to three substances of nitrous oxide, agmatine, and glutamic acid. Nitric oxide (NO) is a highly reactive and unstable substance with a very short half-life, which is a potent vasodilator acting via the intracellular second-messenger cGMP. In healthy humans, l-arginine induces peripheral vasodilation and inhibits platelet aggregation due to an increased NO production [48], but acute L-arginine supplementation does not increase plasma concentration of NOx in healthy individuals with normal plasma concentrations of ADMA [49].

Agmatine, a product of arginine decarboxylation, influences multiple physiologic and metabolic functions. The findings suggest that AGM elevated the synthesis and levels of cAMP, thereby mimicking the effects of caloric restriction with respect to metabolic reprogramming [50]. The effects of injected agmatine in animals include anticonvulsant-, antineurotoxic-, and antidepressant-like actions [51]. Agmatine has neuroprotective effects [52].

The effectiveness of arginine in reducing seizure frequency may be due to the increase of glutamate level, rather than the direct intervention of arginine itself, as the participants of the present study, in the two groups of glutamic acid and arginine, show no significant difference in terms of age (Table 3) and sex distribution (Table 2). Two products of arginine: glutamic acid and agmatine may be involved for anticonvulsant effects of arginine.

Concerning lysine, no significant decrease (*P* = 0.720) was observed in reducing seizure frequency in uncontrolled epilepsy patients (13%). A study has reported that the intraperitoneal injection of lysine reduces the severity of PTZ-induced seizures [53, 54]. As for the present study, mode of lysine administration was oral. In another study on rats with oral lysine administered, similar results were reported [19].

In this study we could not evaluate the effect of charged amino acid on different types of epilepsy due to our limited cases (Table 4).

## 5. Conclusion

Administration of arginine and glutamic acid can be effective in reducing the seizure frequency of uncontrolled epilepsy patients, without triggering considerable complications.

## Figures and Tables

**Table 1:** The rate of seizure attacks (one week before starting amino acid, first week after starting, second week, and ratio of second week to before starting).

Amino acid	Number	Before starting	First week	Second week	Ratio before/second	*t*-test	*P* value
Arginine	10	23.6 ± 16.2	21.6 ± 14.2	11.2 ± 11.5	0.47	3.139	0.013
Glutamic acid	11	31.3 ± 36.2	31.4 ± 36.5	18.54 ± 21.1	0.59	2.646	0.027
Lysine	11	11.4 ± 9.9	12.2 ± 13.7	10 ± 15.5	0.87	0.368	0.720

**Table 2:** The rate of sex of patients.

Sex/amino acid	Arginine	Glutamic acid	Lysine	Total
Male	8	5	7	20
Female	2	6	4	12

Total	10	11	11	32

*P* = 0.28.

**Table 3:** Mean age of patients.

Amino acid/mean age	Number	Mean of ages
Arginine	10	16.2 ± 10.4
Glutamic acid	11	12.27 ± 12.8
Lysine	11	13.27 ± 10.7

*P* = 0.7.

**Table 4:** The type of seizures in this study.

Amino/type of seizure	Tonic-clonic	F-G	Atypical	Lennox-Gastaut	CPS	Myoclonic	Total
Arginine	3	3	1	1	2	0	10
Glutamic acid	0	3	2	2	1	3	11
Lysine	2	2	1	2	2	2	11

Total	5	8	4	5	5	5	32

F-G: secondary generalized, CPS: complex partial seizure, and LGS: Lennox-Gastaut syndrome.
